# Dr. Ireland on Fungus Hæmatodes

**Published:** 1811-04

**Authors:** Joshua Ireland

**Affiliations:** Carlisle


*To the Editors of the Medical and Physical Journal.*

Gentlemen,

By consulting Mr. Wardross's treatise on fungus hæmatodes, you will find the case of a boy whose eye was affected with this complaint, communicated to him by Mr. George Bell, surgeon in Edinburgh. The frequency of failure in operating on such cases, induces me to lay before your numerous readers the result of that and two others, as encouraging some degree of hope that a complaint, which has hitherto been considered desperate, may be relieved, if not cured, by the surgeon's art.

The boy, Richard Green, to whom Mr. Bell alludes, lives in Carlisle. In the end of the year 1808, he applied to me in consequence of this complaint of his eye. At that time the tumour appeared to be of the size of a large sized plum; the tarsi were also affected with swelling and discharge, and every trace of the transparency of the eye was lost. It had become of an irregular tuberculated shape, and as the excruciating pain deprived the boy of rest, impaired his appetite, and was attended with great emaciation and loss of strength; the boy, though nearly seven years of age, not appearing more than four or five, I advised the extirpation of the eye as the only probable means of recovery. To this his parents consented, and the following was the method I pursued. Conceiving that the tarsi might have partaken of the disease, I made a semicircular incision through them, nearly in a line with the edges of the orbit, and then with a dissecting hook, (not quite so much bent as that in common use) pushed to the bottom of the orbit, so as to act like a lever, I dissected out every part contained in it, so that nothing was left but the periosteum. I next filled the vacuity with lint dropped in tinct. myrrh, and endeavoured to draw the divided edges of the eye-lids together by means of ligatures, leaving a small space for the discharge of matter and the extraction of the lint. The parts were kept in this situation for two days by proper compresses and the aid of an assistant, assistant, and without any material symptom occurring, the patient completely recovered in about three weeks. The cicatrix is scarce perceptible, and the boy enjoys a good state of health.

I forgot to mention that there did not appear any perceptible change in the state of the optic nerve. I forbear to make any remark on this singular complaint, until I have detailed the other cases.

JOSHUA IRELAND, M.D.

*Carlisle, Feb.* 15, 1811.

